# Retraction Note: High doses of garlic extract significantly attenuated the ratio of serum LDL to HDL level in rat-fed with hypercholesterolemia diet

**DOI:** 10.1186/s13000-016-0557-4

**Published:** 2016-11-02

**Authors:** Tahereh Ebrahimi, Behnoosh Behdad, Maryam Agha Abbasi, Rahman Ghaffarzadegan Rabati, Amir Farshid Fayyaz, Vahid Behnod, Ali Asgari

**Affiliations:** 1 Department of Agricultural Biotechnology, Sari Agricultural Sciences and Natural Resources University, Sari, Iran; 2 Shahid Beheshti University of Medical Sciences, Tehran, Iran; 3 Department of Biochemistry, College of Science, Kurdistan Science and Research Branch, Islamic Azad University, Sanandaj, Iran; 4 Danesh Pathobiology Laboratory, Tehran, Iran; 5 Shahid Abbas Abdollahi, Molecular Biology Research Center of Shahid Mahallati Hospital, Tabriz, Iran; 6 Department of Legal Medicine, AJA University of Medical Sciences, Tehran, Iran; 7 Baqiyatallah University of Medical Sciences, Tehran, Iran; 8 Department of Infectious Diseases, AJA University of Medical Sciences, Tehran, Iran

## Retraction

The Editor-in-Chief and Publisher have retracted this article [1] because the scientific integrity of the content cannot be guaranteed. An investigation by the Publisher found it to be one of a group of articles we have identified as showing evidence suggestive of attempts to subvert the peer review and publication system to inappropriately obtain or allocate authorship. This article showed evidence of plagiarism (most notably from the articles cited [2–4]) and authorship manipulation.
